# Source-based neurofeedback methods using EEG recordings: training altered brain activity in a functional brain source derived from blind source separation

**DOI:** 10.3389/fnbeh.2014.00373

**Published:** 2014-10-22

**Authors:** David J. White, Marco Congedo, Joseph Ciorciari

**Affiliations:** ^1^Centre for Human Psychopharmacology, School of Health Sciences, Swinburne University of TechnologyHawthorn, VIC, Australia; ^2^Grenoble Images Parole Signal Automatique (Gipsa-lab), CNRS and Grenoble UniversityGrenoble, France; ^3^Brain and Psychological Sciences Research Centre, School of Health Sciences, Swinburne University of TechnologyHawthorn, VIC, Australia

**Keywords:** neurofeedback, EEG, blind source separation, BSS, theta

## Abstract

A developing literature explores the use of neurofeedback in the treatment of a range of clinical conditions, particularly ADHD and epilepsy, whilst neurofeedback also provides an experimental tool for studying the functional significance of endogenous brain activity. A critical component of any neurofeedback method is the underlying physiological signal which forms the basis for the feedback. While the past decade has seen the emergence of fMRI-based protocols training spatially confined BOLD activity, traditional neurofeedback has utilized a small number of electrode sites on the scalp. As scalp EEG at a given electrode site reflects a linear mixture of activity from multiple brain sources and artifacts, efforts to successfully acquire some level of control over the signal may be confounded by these extraneous sources. Further, in the event of successful training, these traditional neurofeedback methods are likely influencing multiple brain regions and processes. The present work describes the use of source-based signal processing methods in EEG neurofeedback. The feasibility and potential utility of such methods were explored in an experiment training increased theta oscillatory activity in a source derived from Blind Source Separation (BSS) of EEG data obtained during completion of a complex cognitive task (spatial navigation). Learned increases in theta activity were observed in two of the four participants to complete 20 sessions of neurofeedback targeting this individually defined functional brain source. Source-based EEG neurofeedback methods using BSS may offer important advantages over traditional neurofeedback, by targeting the desired physiological signal in a more functionally and spatially specific manner. Having provided preliminary evidence of the feasibility of these methods, future work may study a range of clinically and experimentally relevant brain processes where individual brain sources may be targeted by source-based EEG neurofeedback.

## Introduction

The activity-dependent nature of neuroplasticity in the brain has highlighted the potential for manipulations of brain activity in enhancing our understanding of brain processes, but also treating clinical conditions (Cramer et al., 2011). A number of methods exist which apply external stimulation or manipulations to alter brain activity, these include pharmacological interventions, electrical stimulation methods (e.g., deep brain stimulation, transcranial direct current and alternating current stimulation) and Transcranial Magnetic Stimulation (TMS). Unlike these external stimulus driven methods, neurofeedback offers a non-invasive technique capable of manipulating endogenous brain activity. A developing literature supports the use of neurofeedback in the treatment of a range of clinical conditions, particularly ADHD (Arns et al., 2013, 2014) and epilepsy (Sterman and Egner, 2006; Tan et al., 2009). In addition, experimental neurofeedback enables the study of brain activity as the independent variable, providing a powerful method for studying the functional significance of endogenous brain activity (Weiskopf, 2012).

Traditional EEG neurofeedback methods typically utilize a small number of active electrodes on the scalp. Scalp EEG at a given electrode site reflects a linear mixture of activity of multiple brain sources and artifacts, with skull and other tissue having a spatial smearing effect (Congedo et al., 2008). With this in mind, sources optimally aligned and in closer proximity to the scalp electrode represent a greater proportion of the observed activity, but far from the entirety of the observed signal. Thus, traditional neurofeedback methods training single electrode sites are likely influencing multiple brain regions and processes. It is therefore not surprising that training methods using a single scalp site influence large scale EEG dynamics beyond the training frequency and site (for example, Egner et al., 2004). Additionally, as the observed signal reflects multiple brain processes, it has also been suggested that this may impede the ability to acquire control over the feedback signal. This point was highlighted by Philippens and Vanwersch (2010), who demonstrated learned sensory-motor rhythm (SMR) enhancement in four sessions of neurofeedback in non-human primates using intracranial recordings. These authors stressed the ability to acquire control in such a short training period may have partially been a result of the increased spatial resolution, and reduced influence of EMG artifact. Given these limitations of traditional EEG neurofeedback methods, a number of more spatially and functionally specific neurofeedback techniques have been explored. The major development in this area is fMRI-based neurofeedback (Yoo and Jolesz, 2002; Weiskopf et al., 2003, 2004), but also includes spatially specific MEG-based neurofeedback (Florin et al., 2014).

In light of these emerging neurofeedback methods, efforts to develop methods which maximize the functional and spatial specificity of EEG-based neurofeedback techniques remain pertinent given the comparative availability and affordability of such technology, and the capacity to directly target endogenous electrophysiological activity (cf. fMRI methods based on the BOLD response). While source-based EEG neurofeedback using source localization methods has been demonstrated (Congedo et al., 2004), offering potential for an improved spatial precision of a training region, these methods remain limited by the susceptibility of source localization methods to artifacts, the inability to isolate neighboring but functionally separate sources, and the spatial precision offered. Perhaps for these reasons, the capacity for learned regulation using these methods has been inconsistently shown (Maurizio et al., 2014). Blind Source Separation (BSS) is a group of processing techniques which seek to identify source activity from a mixed signal. These methods have been employed in a variety of fields including speech processing (Jang et al., 2002), face recognition (Yuen and Lai, 2002), wireless communication (Van Der Veen et al., 1997), radar applications (Fiori, 2003), and with a range of biomedical signals (James and Hesse, 2005). The blind nature of these methods has facilitated such widespread applicability, where no knowledge of the source activity or mixing process is required. Given the properties of scalp EEG, viewed as an instantaneous linear mixture of multiple brain sources and artifacts as a result of volume conduction, BSS was identified as a method suited to EEG signal processing (Makeig et al., 1996), and has subsequently seen widespread use in EEG research both in the identification and removal of artifacts (Vigário, 1997; Delorme et al., 2007; Romero et al., 2008), and the exploration of functionally and spatially distinct brain sources (Makeig et al., 2004; Onton et al., 2005, 2006; Congedo et al., 2008; Kopřivová et al., 2011). It has recently been proposed that BSS methods may have important advantages in multi-channel neurofeedback beyond those methods based on source localization. Specifically, BSS-based neurofeedback may address the limitations of previous source-based neurofeedback methods by offering enhanced spatial and functional specificity of the training substrate, while being less susceptible to artifacts and noise, and being computationally inexpensive (Congedo and Joffe, 2007; Grandchamp and Delorme, 2009).

Neurofeedback based on sources derived from signal processing methods such as BSS may be ideally suited to isolating a spatially and functionally distinct source, which may be less susceptible to common artifacts, representing significant advantages over traditional methods. Further, given the prominence of BSS-based signal processing methods in the field of cognitive neuroscience, particularly with EEG, demonstrating the “trainability” of functional sources derived from these methods with neurofeedback may open future investigations to study the functional significance of identified sources via trained perturbation of this activity. To this end, the present investigation explores the capacity to learn enhanced activity on a BSS-derived source derived from functional brain activity during completion of a complex cognitive task.

## Materials and methods

### Participants and experimental design

Four healthy right-handed adult volunteers aged 24–38 years old participated in the study (1 female). Written informed consent was obtained from all participants, with all procedures carried out in accordance with the Swinburne University Human Research Ethics Committee. Participants underwent 20 sessions of neurofeedback over the course of 7 weeks. In addition, three assessment sessions were completed across the course of the neurofeedback period, one at baseline, one after 10 sessions, and a final assessment after the 20 sessions. Beyond the data from the baseline assessment session used to isolate individual sources for neurofeedback, these assessment sessions will not be further discussed in the present report.

### BSS-based neurofeedback

The linear BSS problem can be defined as:

(1) x(t)=As(t)

where *x*(*t*) is the observed data and *s*(*t*) the underlying source signals, *A* is a time-invariant mixing matrix. Following matrix algebra, estimated source activity is thus given by:

(2) $$\hat{s}(t)=Bx(t)$$

where *B*, known as the separating or demixing matrix, is the pseudo-inverse of *A*. In this way, reconstructed source activity is given by multiplying the separating matrix by the observed data. This BSS model assumes that observed signals are an instantaneous linear mixture of underlying sources (Cardoso, 1998). These methods typically seek the separating and mixing matrices through cancellation of second order or higher order statistics, seeking maximally independent sources.

When applied to EEG data, *x*(*t*) above is an *n* (electrodes) by *t* (time points) matrix of observed scalp EEG, the mixing matrix (*A*) describes the relative weights with which each source projects to the scalp, and the separating matrix (*B*) obtains the estimated brain source activity, in the form of arbitrarily scaled reconstructed source time-series, when multiplied by the observed EEG. It follows that the estimated activity, or time-series, of a single source of interest $\left({\hat{s}}_{i}(t)\right)$ is obtained by:

(4) $${\hat{s}}_{i}(t)=B_{i}x(t)$$

that is, by multiplying the observed scalp data by the vector of weights (*B_i_*) from the separating matrix which corresponds to the source of interest. The separating matrix can thus be conceived as a spatial filter, used to estimate source activity. In the context of real-time BSS-neurofeedback, online multiplication of scalp EEG by the vector of the separating matrix corresponding to the target training source will obtain the source time-series. The major issue with such an approach is identifying and obtaining a stable estimate of the spatial filter for the training source. The method adopted in order to achieve this in the present experiment was to base the training on a robust task-related source identified in a group BSS analysis, from which the most closely related individual source was sought.

#### Determining individual neurofeedback sources

The functional brain source selected for neurofeedback training was based on a previously reported BSS-derived source including medial-temporal lobe (MTL) and parietal lobe regions in which spatial memory performance was associated with source theta oscillatory activity (White et al., 2012). As part of this study, EEG data during completion of a spatial navigation task was analyzed using a BSS method known as Approximate Joint Diagonalization of Cospectral matrices (AJDC; Congedo et al., 2008). Using this method, a source was identified which demonstrated significantly increased theta oscillatory activity during navigation. Within a sample of 25 healthy adults, greater theta power within this source, localized to MTL and parietal regions using sLORETA (Pascual-Marqui, 2002), was associated with better task performance.

As part of the neurofeedback protocol, each participant required individually determined weights corresponding to the BSS component showing the strongest correlation with the group MTL—parietal theta source identified in White et al. (2012) during completion of this same task. Using identical EEG acquisition and pre-processing routines as that used in White et al. (2012), individual participants’ EEG data during spatial navigation was decomposed using the identical BSS method (AJDC using the same parameters previously reported, using ICoN software, Version 3.1^1^). Source time-series derived from the individual BSS decomposition were then correlated with the group MTL/parietal theta source time-series described in White et al. (2012), with the individual source showing the strongest correlation selected as the feedback source (for all four participants, *r* ≥ ± 0.550). The weights corresponding to this component in the separating matrix were extracted for use as a spatial filter for neurofeedback, using a subset of electrodes which did not compromise the source signal (39–42 electrodes were retained for neurofeedback sessions, from the original 62). Peak theta for the feedback training band was determined as peak power within the 4–8 Hz band via Fast-Fourier Transform of individual source activity during completion of the navigation task.

#### Neurofeedback protocol

All participants underwent 20 neurofeedback sessions across 7 weeks. Each neurofeedback session involved a resting eyes open baseline, then five blocks of training each lasting 4 min. As this study represented a preliminary investigation exploring the feasibility of BSS-based training, no control neurofeedback group was included. Instead, a series of trials were conducted at a follow-up session upon completion of the 20 sessions in which participants were asked to increase or decrease the feedback signal. As the focus of this experiment was the feasibility of learned regulation of BSS-derived source activity, these trials were included to probe for evidence of learned volitional regulation of the target signal. SynAmps^2^ amplifiers and Acquire 4.3 software were used to acquire the EEG data as part of the neurofeedback sessions (Neuroscan Inc., Abbotsford, VIC, Australia). Data acquisition for neurofeedback sessions employed a band-pass filter from 1–50 Hz, with a linked mastoid reference. This was done to ensure consistency with the reference used in the off-line analysis of the previous experiment. A second computer running the Open-ViBE software platform (Renard et al., 2010) provided the on-line processing and feedback required by the neurofeedback paradigm. The set-up made use of the built-in client/server operations available in Scan Acquire 4.3 software; whereby the acquisition system acted as the server which sent acquired data on to the client system (Open-ViBE) via a Local Area Network. An acquisition driver written in C++ facilitated this process within the Open-ViBE platform.

A “Scenario” was developed for each participant within the Open-ViBE software which applied a processing chain to generate the feedback signal, before providing visual feedback to the participant with minimal delay. The Scenario for each participant applied the individually defined spatial filter to the incoming EEG data, generated a ratio of peak theta (*pθ* = peak ± 0.5 Hz) to total theta (*totθ* = 4–8 Hz) for the source time-series, then provided visual feedback. In order to obtain on-line band-power estimates for *pθ* and *totθ* the time-series was first band-pass filtered in the designated frequency range (Butterworth filter, 0.5 dB band-pass ripple), then segmented into 1 s epochs with a 250 ms moving window, the data were then squared and an average calculated for each 1 s epoch. Finally, each estimate was log transformed (ln(*x* + 1)) to minimize deviations from normality (Kiebel et al., 2005). The feedback signal was simply the ratio of these log transformed band-power estimates for peak and total theta (*pθ/totθ*). Electro-oculogram (EOG) artifact remains an important consideration when dealing with theta-band activity. This motivated the use of a ratio, as opposed to absolute power. Eye blink and movement artifact is generally maximal in the delta range, decreasing in a steep and monotonous manner with increasing frequency (Gasser et al., 1985; Hagemann and Naumann, 2001). We reasoned that in using the ratio of peak theta activity relative to total theta, the potential confounding influence EOG artifact would be minimized. For example, it is highly unlikely that EOG artifact would manifest itself as a frequency-specific power increase coinciding with peak theta activity, and much more likely that the presence of EOG artifact would result in broadband theta power increases, largest at the low end of the bandwidth, resulting in little change or a drop in the *pθ/totθ* ratio.

**Figure 1:**
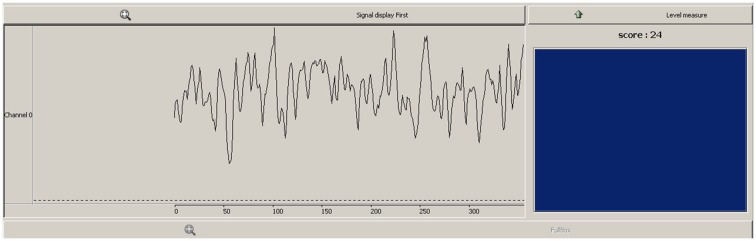
**Feedback viewed by participants during neurofeedback sessions**. Line graph (left) showed continuous feedback as to the *pθ/totθ* ratio level, box and score (right) provided a discrete reward when ratio exceeded a predefined threshold.

The feedback received by the participant contained both continuous and discrete elements (see Figure 1). A scrolling line graph showing the exact ratio level formed the continuous feedback, whilst a reward box flashed blue and registered a point each time the ratio exceeded a predefined threshold. The continuous feedback has the advantage of being easy to interpret for the participant (Weiskopf et al., 2004), whilst the threshold score and blue flash provided a discrete reward, which utilizes the common conception of neurofeedback learning by means of operant conditioning. The importance of discrete feedback in neurofeedback protocol design has recently been emphasized (Sherlin et al., 2011). This threshold was determined by calculating a percentile during the baseline recording of the first neurofeedback session, where 6–12 discrete rewards would be received per minute at baseline levels.

#### Assessment of neurofeedback learning

The capacity to regulate the feedback signal, in the form of neurofeedback learning, forms the primary outcome for this feasibility study. A number of methods have been adopted for operationalizing and quantifying relative success at neurofeedback learning, yet there is little agreement on the most appropriate method (Dempster and Vernon, 2009). In order to demonstrate learned control over the feedback signal, evidence that changes occur beyond baseline levels appears a minimum requirement. In order to first assess this, non-learners were identified with an initial paired samples *t*-test, contrasting mean source *pθ/totθ* ratio scores at all 20 feedback sessions for each participant with the corresponding baseline. Only those to demonstrate significantly elevated source *pθ/totθ* ratio during feedback when contrasted with corresponding baseline were analyzed for evidence of neurofeedback learning. Learning within and across sessions involve desired changes emerging over the course of training, and thus can be plotted as a learning curve. In each of these cases, the presence of learning is demonstrated by the desired increase or decrease in the signal of interest across sessions or over blocks of time within sessions. Within and across sessions learning was assessed by ordinary least squares regression, with the time within training as the predictor (session number for across sessions, and minute from beginning of feedback for within sessions; both 1–20). For both learning analyses, data was normalized with respect to the mean and standard deviation of source *pθ/totθ* ratio during a baseline period. For within sessions learning, mean data was extracted for each minute of neurofeedback at each session, and normalized to the corresponding baseline data for that session. Across sessions learning analysis used the mean source* pθ/totθ* ratio for each session normalized to the baseline period at the first neurofeedback session.

Clearest demonstrations of volitional self-regulation use a series of trials in which the participant is instructed to produce the desired change in signal, contrasted with trials where the opposite change or no change are desired. Neurofeedback for Slow Cortical Potentials (SCP; for a review see Birbaumer, 1999) lends itself to this type of analysis, and self-regulation of positive and negative shifts have been demonstrated in this way (e.g., Schneider et al., 1992). Volitional self-regulation has also been demonstrated with the use of specifically conceived experimental design following LORETA neurofeedback training for enhanced low beta activity in the anterior cingulate gyrus (Congedo et al., 2004). As part of the present exploration, participants completed a follow-up session upon completion of the 20 neurofeedback sessions in which they were asked to increase or decrease the feedback signal in eight randomized blocks of 3-min each (total of four “up” and four “down” trials). A randomization *t*-test (Edgington, 1987) comparing the mean *pθ/totθ* ratio feedback signal obtained in each up trial vs. the mean of each down trial exploits the design of the trials, providing an appropriate assessment of volitional self-regulation of the feedback signal post-training.

A designated exploration of the relationship between ocular artifact and the feedback signal was undertaken offline, using data from the first neurofeedback session for each participant. Power in the delta range (1–3 Hz), averaged across frontal electrode sites, formed a surrogate measure of EOG artifact. For each participant, power estimates were calculated in the same way as those used to derive the source peak theta to total theta ratio, with an additional parallel processing stream calculating frontal delta power. The relationship between the two signals was then assessed by correlating the power estimates for the two signals for the first session in each neurofeedback participant.

#### Assessing the impact of neurofeedback training on navigation performance

Owing to the exploratory nature of the current experiment, the sample size limited the scope for a full exploration of the impact of the neurofeedback intervention, instead focussing on the feasibility of such a protocol. While this prevented the use of traditional statistical analysis of group differences in outcome measures across the intervention period, we briefly describe the trends in behavioral performance on the navigation task from which the neurofeedback source was derived across the neurofeedback training period contrasted with an age and gender matched no-treatment control group. Three assessment points were completed across 7 weeks for both neurofeedback and control participants (pre-treatment baseline, week 4, week 7 (post-treatment)). The difference in these trends, in the form of the gradient of the slope estimated by Ordinary Least Squares from all navigation performance observations for each group, was then contrasted between neurofeedback and control group using a small sample *t*-test for parallelism (Kleinbaum and Kupper, 1978).

## Results

### Exceeding resting levels

**Figure 2:**
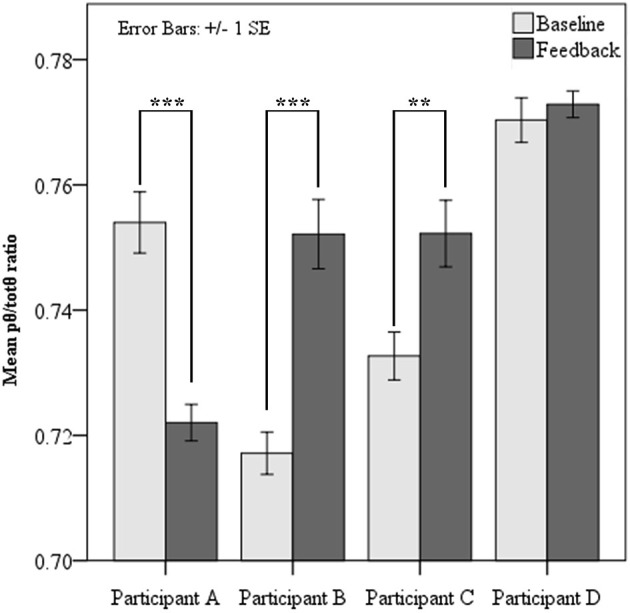
**Mean *pθ/totθ* ratio for feedback source at resting baseline and during feedback for each participant**. Paired *t-*tests showed significantly increased *pθ/totθ* ratio during feedback when compared to baseline for Participants B and C. Participant A showed significantly lower *pθ/totθ* ratio during feedback (*** = *p* < 0.001, ** = *p* < 0.01).

Paired samples *t*-tests revealed two of the four participants demonstrated significantly elevated source *pθ/totθ* during neurofeedback sessions, when contrasted with baseline at each session (see Figure 2 below). Analysis of learning trends was pursued for these two participants only. Surprisingly, one participant showed a significant reduction in the feedback ratio during feedback sessions compared to baseline, while one showed slight non-significant increases from baseline to feedback.

### Neurofeedback learning within and across sessions

**Figure 3:**
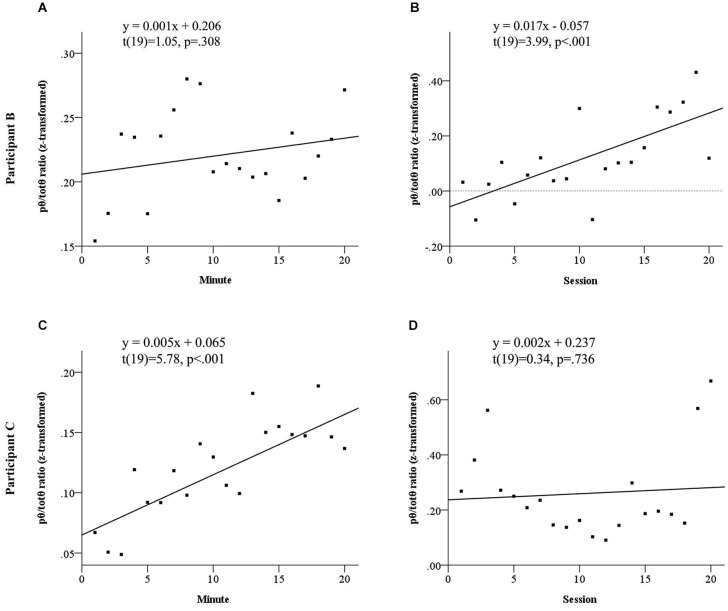
**Learning trends in normalized *pθ/totθ* ratio for feedback source both within and across sessions for Participant B, see panels (A) and (B) and Participant C, see panels (C) and (D)**. Participant B did not show a significant linear increase within sessions **(A)**, however showed significant across sessions learning **(B)**. Participant C showed significant within sessions learning **(C)**, but no linear increases across sessions.

Both participants to show elevated source *pθ/totθ* during neurofeedback sessions demonstrated evidence of neurofeedback learning. Figure 3 summarizes the results of linear regression analyses probing neurofeedback learning within and across sessions for these two participants. This learning emerged across sessions for Participant B, but within sessions for Participant C. Participant B appeared to demonstrate a sharp within sessions learning trend in the first half of each session, before significantly dropping away.

### Volitional control of source *pθ/totθ* ratio

**Figure 4:**
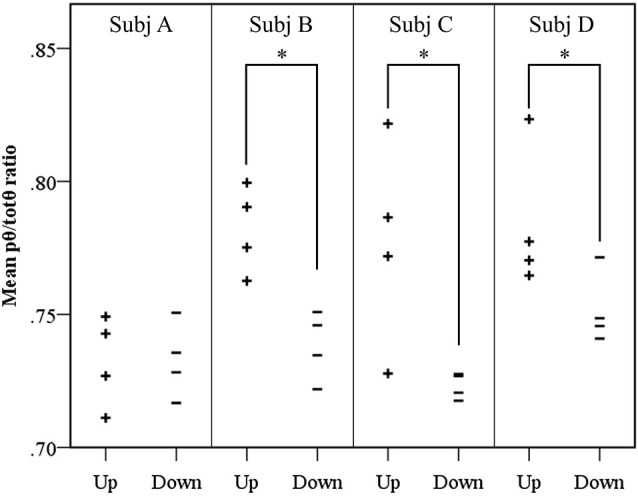
**Results of volitional control trials conducted after neurofeedback training**. For each participant, the mean source *pθ/totθ* ratio for each trial is shown. A significantly elevated ratio was observed in up trials, contrasted with down trials using a randomization *t*-test, for three of the four participants, where * = *p* < 0.05.

Results of the follow-up session exploring volitional control of the feedback signal corroborated findings of the neurofeedback learning analyses. Figure 4 shows Participants B and C again demonstrated significantly greater *pθ/totθ* source activity during the “up” trials than the “down” trials (Participant B: *t* = 4.19, *p* = 0.0143; Participant C: *t* = 2.75, *p* = 0.0143). In addition, the findings from these trials suggested Participant D obtained some level of volitional control over the signal that did not translate into neurofeedback learning during the training period (Participant D: *t* = 2.15, *p* = 0.0429).

### Ocular artifact

The selection of a ratio feedback signal (*pθ/totθ*) was motivated by a desire to minimize the influence of artifacts in neurofeedback sessions. However, the potential influence of ocular artifact was explored offline, correlating delta power estimates at frontal electrode sites with the feedback signal during neurofeedback. Results of this analysis suggested minimal relationship between the source feedback ratio and frontal delta power for each participant (Participant A: *r* = 0.004; Participant B: *r* = −0.002; Participant C: *r* = 0.115; Participant D: *r* = 0.043). As the maximum value, observed in Participant C corresponds to approximately 1% of variance explained, it appears unlikely that any participant experienced the capacity to achieve the desired signal increases through increasing the presence of ocular artifact.

### Navigation performance

**Figure 5:**
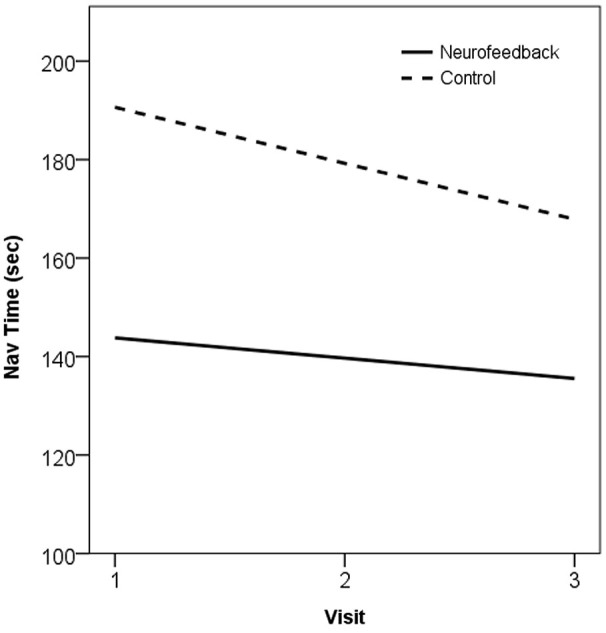
**Plot of trend in navigation performance across the training period, as the OLS line of best fit, in neurofeedback and no-treatment control groups**. 1 = Baseline, 2 = Mid-training (week 4), 3 = Post-training (week 7).

Trends in behavioral performance on the navigation task assessed across the training period showed evidence of improvement in both neurofeedback and control groups. The two groups showed large baseline differences in performance, and evidence of practice effects across repeated assessments, but the trend across the training period did not significantly differ between the groups (*t*_(20)_ = −0.27,* p* > 0.05). These trends, in the form of the gradient of the slope estimated by Ordinary Least Squares (β), are plotted for performance on the navigation task in Figure 5.

## Discussion

The current study described the implementation of a neurofeedback paradigm using spatial filtering of scalp EEG to obtain ongoing activity of a BSS component derived from functional brain activity. The study aimed to explore the feasibility of training enhanced theta using this BSS-based neurofeedback. Results showed evidence of learned augmentation of source peak theta activity in 50% of the neurofeedback sample, providing preliminary evidence in support of the feasibility of BSS-based neurofeedback. Beyond this evidence of neurofeedback learning trends, the study also demonstrated volitional control of the feedback signal upon completion of the neurofeedback training period in three of the neurofeedback participants. No differences in behavioral performance were observed for the neurofeedback group on the navigation task from which the training source was derived, when compared to an age and gender matched no-treatment control group. Thus, the findings of this study suggest learned regulation of oscillatory activity derived from a BSS component represents a plausible line of inquiry for future research.

### Non-learners and study limitations

In the present study, two participants failed to show evidence of learned control over peak theta activity beyond baseline levels. An important consideration in analyzing neurofeedback learning is that not all those exposed to training will gain significant control over the feedback signal. Previous explorations of neurofeedback learners and non-learners suggest that as much as half of those participating in neurofeedback training may not demonstrate significant learning (Weber et al., 2011). In line with this, 50% of the present sample belongs to this non-learner group, while 25% showed no evidence of volitional control of the feedback signal upon completion of the training. This aspect of neurofeedback training is relatively unexplored, and the characteristics and predictors of learners and non-learners is an area requiring further empirical exploration.

One possible explanation for the difficulty in training source peak theta activity in these non-learners is that the source contained a spectral profile which included a low alpha component. The low alpha band shows basic attentional correlates and desynchronizes in response to task demands (for a review, see Klimesch, 1999). Individually determined peak theta for the two non-learners was slightly higher than in the other two participants. As the training frequency for these participants was adjacent to the low alpha band, spectral power associated with alpha activity may have leaked into peak theta estimates. Indeed, the functional source on which the feedback was based showed a clear alpha peak in the original group data around 10–11 Hz during a resting baseline (see White et al., 2012). Thus, a reduction in low alpha activity during training could have confounded efforts to increase peak theta activity for these non-learners during neurofeedback trials, as the increased attention associated with the training periods would be reasonably expected to reduce low alpha activity. Whilst the BSS-derived training source is argued to be functionally and spatially specific, this does not preclude spectral activity across multiple frequencies. Thus, the lack of learning in these participants may have been a result of contamination of the desired peak theta signal from the adjacent low alpha band. This may be particularly relevant for Participant A, who showed significant reductions in the feedback signal during feedback when contrasted with baseline. The findings of the present study further emphasize the need to account for fluctuations in a number of frequency bands beyond the training band in neurofeedback research.

As this study represents a preliminary investigation into BSS-based neurofeedback, interpretation of findings remain limited by the design implemented and small sample size. Neurofeedback research is increasingly utilizing experimental designs which incorporate control conditions to allow for stronger evidence of efficacy and specificity, but also the feasibility of learning. In examining efficacy and specificity of clinical and experimental neurofeedback protocols, the use of non-contingent feedback, variable feedback contingencies (e.g., Hoedlmoser et al., 2008), or alternate target bands for neurofeedback control groups have been used to minimize concerns to do with comparable therapist contact, placebo effects, and other non-specific effects of training. Having provided this preliminary evidence of the feasibility of training a BSS-based source, future work may further explore the validity and potential applications of neurofeedback training using controlled designs which can target sources identified from functional or resting state brain processes.

### Applications of BSS-based neurofeedback

Developing neurofeedback methods are increasingly refining the spatial and functional specificity with which the training neural substrate can be targeted. Using a BSS-derived source as the feedback source offers advantages in this respect, and the findings of the present study support the feasibility of such methods. Thus, future applications of BSS-based neurofeedback are only limited by the extent to which a stable estimate of the target source can be obtained. As BSS-derived sources can be identified from functional or resting activity, future research can extend the present work by applying neurofeedback protocols based on BSS components based on manipulating functional activity such as that described herein, or training problematic BSS components identified in clinical applications. The potential for clinical applications of BSS-based neurofeedback has recently been explored by Kopřivová et al. (2013), who tested neurofeedback training of a medial frontal EEG source identified as showing abnormally elevated low-frequency activity in Obsessive-Compulsive Disorder patients compared to healthy controls. This BSS-based neurofeedback training was associated with greater clinical improvement than a sham feedback control group and non-significant trends towards a shift in the trained frequency band, however, clinical improvement was not associated with EEG changes. In demonstrating the capacity for regulation of a task-derived BSS source, this study supports the use of BSS-derived sources in neurofeedback applications, as task-related decompositions such as that used to derive the feedback source herein can be considered stable enough to target outside the context of the specific task from which they were based. As such, experimental BSS-based neurofeedback may train functionally and spatially isolated brain sources, facilitating the study of these sources as the independent variable, in turn providing a powerful method for studying the functional significance of functionally and spatially isolated endogenous brain processes. Using BSS-based neurofeedback may enhance the success of neurofeedback protocols, reducing the influence of artifacts, and providing optimal conditions for training of the target activity.

## Conclusions

The research described herein builds upon the increasing use of BSS methods in the study of brain function. Adopting BSS methods across a range of methods for recording brain activity, including fMRI and EEG, has offered novel insights into brain function (eg. Greicius et al., 2004; Onton et al., 2005). These findings provide preliminary evidence of the feasibility of source-based neurofeedback training derived from BSS, future work may explore further validation and potential applications of BSS-based neurofeedback training, targeting sources identified from functional or resting state brain processes.

## Conflict of interest statement

The authors declare that the research was conducted in the absence of any commercial or financial relationships that could be construed as a potential conflict of interest.

## Footnotes

^1^http://sites.google.com/site/marcocongedo/software/icon
